# Supplementary material for: Photonic machine learning with on-chip diffractive optics
Source: Nat Commun. 2023 Jan 5;14:70. doi: 10.1038/s41467-022-35772-7 (PMC9814266; doi:10.1038/s41467-022-35772-7)
Supplement: Supplementary file 1 — Supplementary Information [file 41467_2022_35772_MOESM1_ESM.pdf]

Supplementary Information for

Photonic machine learning with on-chip diffractive optics

Tingzhao Fu[1], Yubin Zang[1], Yuyao Huang[1], Zhenmin Du[1], Honghao Huang[1], Chengyang Hu[1], Minghua Chen[1], Sigang Yang[1], Hongwei Chen[1]✉

[1]Beijing National Research Center for Information Science and Technology, Department of Electronic Engineering, Tsinghua University, Beijing, 100084, China
✉email: chenhw@tsinghua.edu.cn

Supplementary Note 1: On-chip DONN model
Supplementary Note 1.1: On-chip electromagnetic propagation model
Supplementary Note 1.2: Forward and error backward propagation (FEBP)
Supplementary Note 1.3: Neuron-mapping process

Supplementary Note 2: Numerical calculation
Supplementary Note 2.1: Iris flower classifier
Supplementary Note 2.2: Handwritten digit classifier

Supplementary Note 3: Numerical analysis of phase errors

Supplementary Note 4: On-chip DONN-system: fabrication and measurement
Supplementary Note 4.1: Device fabrication of on-chip DONN-I1 and DONN-I3
Supplementary Note 4.2: Experimental demonstration of on-chip DONN-I1 and DONN-I3
Supplementary Note 4.3: Device fabrication of on-chip DONN-M3

Supplementary Note 5: Compensation analysis of DONN errors
Supplementary Note 5.1: System error compensation for the Iris flower classifier
Supplementary Note 5.2: System error compensation for the Handwritten digit classifier

Supplementary Note 6: Algorithm compensation
Supplementary Note 6.1: Parameters of the algorithm compensation process
Supplementary Note 6.2: Pseudo code of the optimization process
Supplementary Note 6.3: Optimization flow chart
Supplementary Note 6.4: Brief introduction of the compensation algorithm

Supplementary Note 7: On-chip DONN computational speed and power consumption
Supplementary Note 7.1: Computation speed
Supplementary Note 7.2: Power consumption

Supplementary Note 8: Comparison of partial performances of different optical neural networks (ONNs)

Supplementary Note 9: Comparison of on-chip DONN-I3 with other research works and commercial products in terms of throughput and operational power consumption

Supplementary References

Supplementary Note 1: On-chip DONN model

Supplementary Note 1.1: On-chip electromagnetic propagation model

On-chip electromagnetic propagation (OEP) model is the first part and prerequisite of the on-chip DONN model. Based on the OEP model, the parameters of on-chip DONNs can be obtained by pre-training in advance. We use the OEP model to approximately calculate the propagation of light in a 2.5-dimensional (2.5D) variational finite-difference time-domain (FDTD) solver (Lumerical Mode Solution commercial software), in this way, when light propagates a certain distance in a slab waveguide, the amplitude and phase correction factors η and $\Delta\phi$ for the modified Huygens-Fresnel principle in Eq. (1) can be determined. Fig. S1c and Fig. S1d indicate the amplitude and phase distribution of the input signal waveform after 250 μm propagation in the slab waveguide (thickness is 220nm, width is 300μm) based on the modified OEP model and 2.5D variational FDTD, respectively. The calculated results of the two models for the electric field propagation of the same input signal (Fig. S1a and Fig. S1b) are highly consistent.

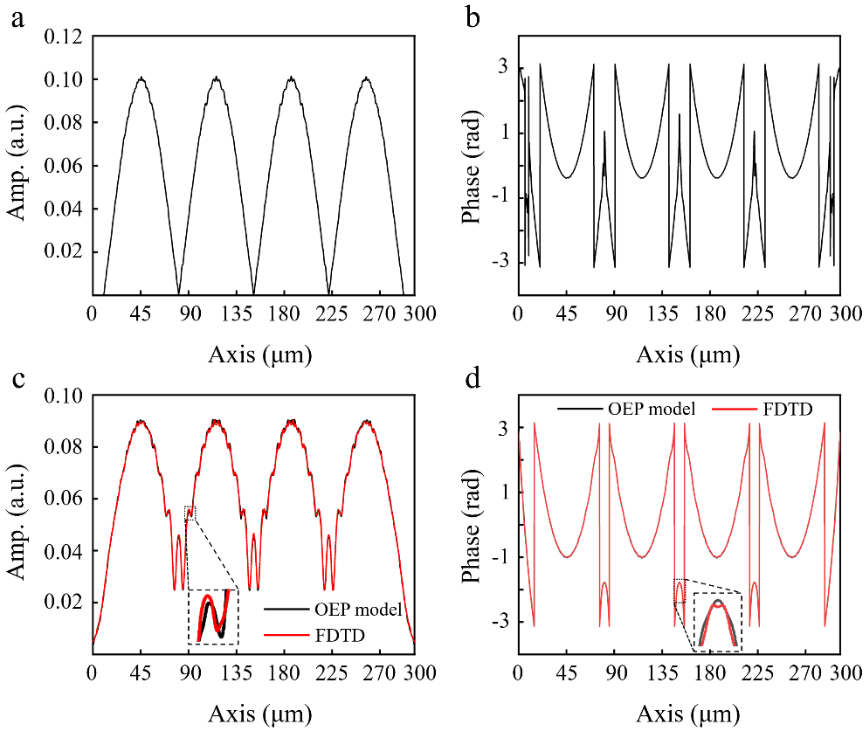

Supplementary Figure 1. Comparison between the on-chip electromagnetic propagation model and the 2.5D variational FDTD calculation results. a, b The field intensity and phase distribution of the input signal, respectively. **c, d** The field intensity and phase distribution of the input signal propagating 250 μm later in a slab waveguide, the results are calculated by the modified OEP model Eq. (1) (black line) and 2.5D variational FDTD (red line), respectively. where, the unit of the normalized power 'a.u.' is the abbreviation of 'arbitrary unit'.

Supplementary Note 1.2: Forward and error backward propagation (FEBP)

The forward propagation process can be expressed by Eq. (S-1):

$$\begin{cases} n_{i,p}^{m} = w_{i,p}^{m} \cdot T_i^{m} \cdot u_i^{m} \\ u_i^{m} = \sum_k n_{k,i}^{m-1} \\ T_i^{m} = A_i^{m} \cdot \exp(j\varphi_i^{m}) \end{cases} \quad \text{(S-1)}$$

where i represents a neuron of the m-th layer, n_i^m is the output function of the i-th neuron, w_i^m is the function of electromagnetic propagation, p refers to a neuron of the next layer that is connected to neuron i by optical diffraction, u_i^m is the input wave to the i-th neuron of layer m, T_i^m is the transmission coefficient of the i-th neuron of layer m, A_i^m and φ_i^m are the amplitude and phase factors, here $A_i^m \approx 1$ [1].

The error backward propagation is realized by the gradient descent algorithm. By assuming that the on-chip DONN consists of M layers (excluding the input and output layer), then the loss function (L) can be defined as Eq. (S-2):

$$\begin{cases} L\left(\varphi_i^m\right) = \dfrac{1}{N}\sum_k \left(\dfrac{S_k^{M+1}}{\sum_k S_k^{M+1}} - Tar_k^{M+1}\right)^2 \\ S_i^{M+1} = \left|u_i^{M+1}\right|^2 \\ u_i^{M+1} = \sum_k n_{k,i}^M \end{cases} \tag{S-2}$$

where $L(\varphi_i^m)$ is the loss function, S_i^{M+1} is the detecting intensity of the output areas, ${T_{ar}}_i^{M+1}$ is the target intensity of the output areas, N refers to the number of measurement points at the output detected area. Therefore, the problem of the optimization for an on-chip DONN can be summarized as $\min L(\varphi_i^m)$, $0 < \varphi_i^m \leq 2\pi$. The gradient calculation formula of the loss function is depicted in Eq. (S-3):

$$\frac{\partial L}{\partial \varphi_i^m} = \frac{4}{N}\sum_k \left(\frac{S_k^{M+1}}{\sum_k S_k^{M+1}} - Tar_k^{M+1}\right) \cdot \frac{\sum_k S_k^{M+1} - S_k^{M+1}}{\left(\sum_k S_k^{M+1}\right)^2} \cdot \mathrm{Real}\left\{\left(u_k^{M+1}\right)^* \cdot \frac{\partial u_k^{M+1}}{\partial \varphi_i^m}\right\} \tag{S-3}$$

where u_k^{M+1} quantifies the gradient of the complex-valued optical field at the output layer with respect to the phase values of the neuron in the previous layers, $m \leq M$. Furthermore, In Eq. (S-3), $\frac{\partial u_k^{M+1}}{\partial \varphi_i^m}$ can be calculated by Eq. (S-4):

$$\frac{\partial u_k^{M+1}}{\partial \varphi_i^{m=M-L}} = j \cdot T_i^{M-L} \cdot u_i^{M-L} \cdot \sum_{k_1} w_{k_1,k}^M \cdot T_{k_1}^M \cdots\cdots \sum_{k_L} w_{k_L,k_{L-1}}^{M-L+1} \cdot T_{k_L}^{M-L+1} \cdot w_{i,k_L}^{M-L} \tag{S-4},$$

where, $3 \leq L \leq M-1$. So far, the parameters of an on-chip DONN can be obtained through continuous optimization in the training process by the gradient descent algorithm. The forward and error backward propagation can also refer to previous works [1,2].

Supplementary Note 1.3: Neuron-mapping process

Based on the OEP model and FEBP process, all parameters of on-chip DONNs can be obtained. Subsequently, it is significantly important to accurately map the pretrained parameters onto physical structures. In particular, the neuron-mapping process is the most critical and challenging. In this study, silicon-based slots are used to realize the pretrained phase values of neurons. According to the effective medium theory (EMT) and FDTD simulation, the effective refractive index (ERI) of the silicon slot filled with silicon dioxide (SSSD) under different widths can be calculated [1,3]. Here, the thickness of the SSSD is 220 nm, the lattice constant (the center distance between two adjacent SSSDs) of the SSSD is fixed as 500 nm, and the width of the SSSD is set as 200 nm by considering the practical fabrication conditions. Therefore, the ERI of the SSSD is calculated as 2.166 by Eq. (2), which is listed here again, as shown in Eq. (S-5).

$$L_{slot-i} = \frac{\Delta\varphi_i}{(n_{eff} - n_{slab}) \cdot k_0} \tag{S-5}$$

where L_{slot-i} represents the length of the SSSD in the i-th group, n_{eff} represents the ERI of the slot group filled with silicon dioxide through which light passes, n_{slab} represents the ERI of the slab waveguide, $k_0 = 2\pi/\lambda$ is the wavenumber of light propagating in vacuum, $\Delta\varphi_i$ represents the phase delay generated by the i-th slot group filled with silicon dioxide. According to Eq. (S-5), the length of the SSSD can be calculated by the pretrained phase value $\Delta\varphi_i$. Thus, the n_{eff} in Eq. (S-5) should be a fixed value during the pre-training process. However, there is mutual interference between adjacent SSSD [1]. When the pretrained neuron values after being mapped onto the physical structures, the n_{eff} realized by the physical structure unit is different from that in the pre-training stage and varies with the change of SSSD length, which seriously affects the neuron-mapping process. To reduce the impacts of mutual interference between adjacent SSSDs during the neuron-mapping process, a slot group filled with silicon dioxide composed of multiple identical SSSDs is used to approximate a phase value of a neuron. The n_{eff} calculated by the phase delay generated by the slot group with a different number of SSSDs is shown in Fig. S2a. It is evident that when the number of orange-yellow SSSD increases, the ERI (the red triangle) calculated by the phase delay generated by the slot groups tends to be a stable value. In addition, the distance between the input light and the hidden layer (HL) will also affect the accuracy of the neuron-mapping process. As shown in Fig. S2b, when the incident light propagates to the HL for 30 µm, the calculation result of the ERI of the identical SSSD by FDTD is different. When the distance between the input light and the HL increases to 250 µm, the value of the ERI tends to be stable. When the distance further increases to 450 µm, the overall change of the ERI of the SSSD is smaller. Therefore, by a comprehensive consideration, the distance between the input light and the first HL and the distance between adjacent HLs are set to 250 µm in this study. The relevant contents can also refer to the previous work [1].

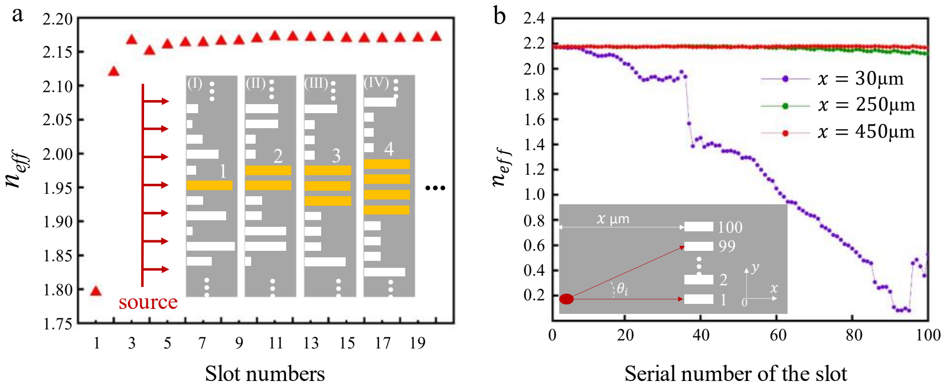

Supplementary Figure 2. Calculation results of effective refractive index (ERI) based on FDTD. a Calculation of the ERI under a different number of identical silicon slots filled with silicon dioxide in a slot group. The incident light source is flat light, and the center distance of the adjacent silicon slot filled with silicon dioxide (SSSD) is 500 nm, the width of the SSSD is 200 nm, the thickness is 220 nm, and the length is 1.964 µm. The length of 1.964 µm is randomly generated. **b** The incident light source is a point light, and the distance between the light source and the HL is 30 µm, 250 µm, and 450 µm, respectively. Among them, the HL consists of 100 identical SSSDs, numbered from 1 to 100. The dots are the ERI values calculated by the phase delay $\Delta\varphi_i$ generated by the light passing through the SSSD of the corresponding sequence number. n_{eff0} is the ERI in the pre-training process, the period of the SSSD is 500 nm, the width of the SSSD is 200 nm, the thickness is 220 nm, and the length is 2 µm.

Supplementary Note 2: Numerical calculation

Supplementary Note 2.1: Iris flower classifier

After the super parameters of on-chip DONNs are determined, the phase values of neurons or other key parameters can be obtained through computer pre-training process based on the on-chip DONN model. Supplementary Table 1 indicates the prediction accuracy of the on-chip DONNs for classification task on the Iris plants dataset, it is evident that with the increase in the number of HLs (m means the number of HLs in the on-chip DONN), the prediction accuracy has not been significantly improved. Therefore, in the process of network design, it is necessary to comprehensively consider the performance of the network, the energy efficiency, and the integration of the chip. Fig. S3a and Fig. S3b are the convergence process of the loss function in the training process of on-chip DONN-I1 and DONN-I3. With the increase of training iterations, the loss curve gradually tends to be stable and converge. Fig. S3c and Fig. S3d show the confusion matrixes of testing results of on-chip DONN-I1 and DONN-I3 on the Iris plants dataset, respectively. In this design, there are 186 neurons on each HL, and their phase values are shown in the form of pixels in Fig. S4. The linear arrangement phase values of each HL are recombined and given in the form of a 6×31 matrix.

Supplementary Table 1 | Prediction accuracy of various on-chip DONNs on the iris blind test sets

DONN with m hidden layers (DONN-Im)	Accuracy
On-chip DONN-I1	86.7%
On-chip DONN-I2	86.7%
On-chip DONN-I3	90%
On-chip DONN-I4	90%
On-chip DONN-I5	90%

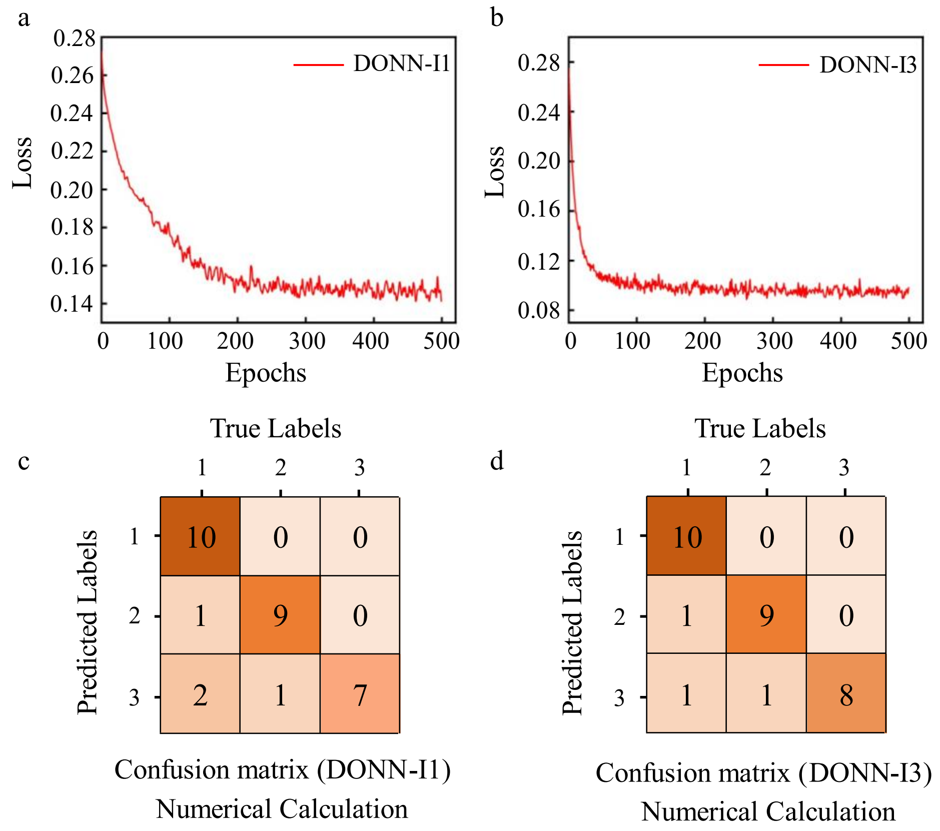

Supplementary Figure 3. Numerical calculation results of on-chip DONN-I1 and DONN-I3. a, b Loss curves of numerical calculations on the training set of the on-chip DONN-I1 and DONN-I3, respectively. **c**, **d** Confusion matrixes for the chosen 30 samples from the Iris plants dataset of the on-chip DONN-I1 and DONN-I3, respectively. Moreover, the labels in the abscissa and ordinate represent the species of the Iris flower, in which 1 represents "Setosa," 2 represents "Versicolor," and 3 represents "Virginia," respectively.

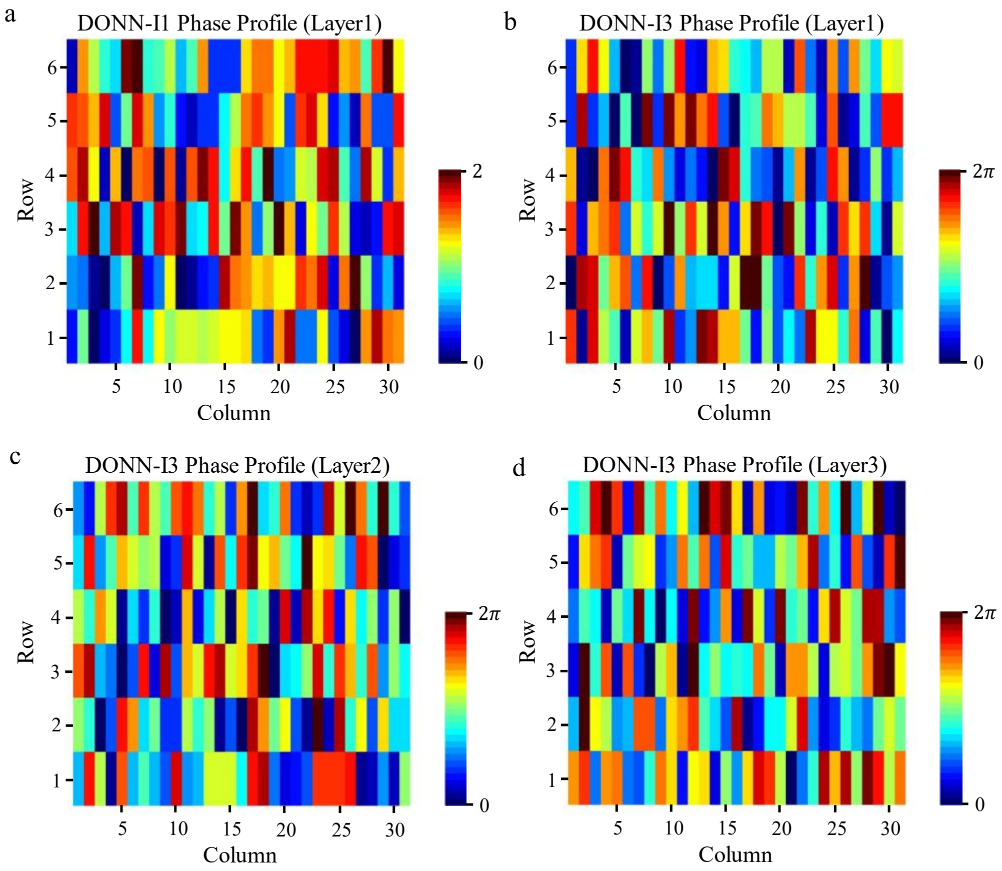

Supplementary Figure 4. Phase profile of each hidden layer of the on-chip DONN-I1 and DONN-I3. The linear phase profile of each hidden layer is converted to a 6×31 pixelated image. **a** Phase profile of the on-chip DONN-I1 hidden layer after training. **b**, **c**, and **d** Phase profiles of the on-chip DONN-I3 hidden layers after training. Moreover, each rectangular pixel in the figure represents a pretrained phase value of a neuron.

Supplementary Note 2.2: Handwritten digit classifier

Supplementary Table 2 indicates the prediction accuracy of the on-chip DONNs for classification task of the MNIST handwritten digit images. Meanwhile, the letter ‘n’ represents the number of HLs in the on-chip DONN (e.g., the DONN-M3 indicates the DONN includes three HLs). Fig. S5a is the convergence process of the loss function in the training process of on-chip DONN-M3. Fig. S5b is the confusion matrixes of blind testing results of on-chip DONN-M3. In this structure, there are 70 neurons on each HL, and phase values of the three HLs are shown in the form of pixels in Fig. S5c (Layer1, Layer2 and Layer3). The linear arrangement phase values of each HL are recombined and given in the form of a 2×35 matrix.

Supplementary Table 2 | The prediction accuracy (numerical calculation) of various on-chip DONNs on the MNIST blind test sets (10000)

DONN with n hidden layers (DONN-Mn)	Accuracy
On-chip DONN-M1	72.6%
On-chip DONN-M2	95.5%
On-chip DONN-M3	96.3%
On-chip DONN-M4	96.5%
On-chip DONN-M5	96.1%

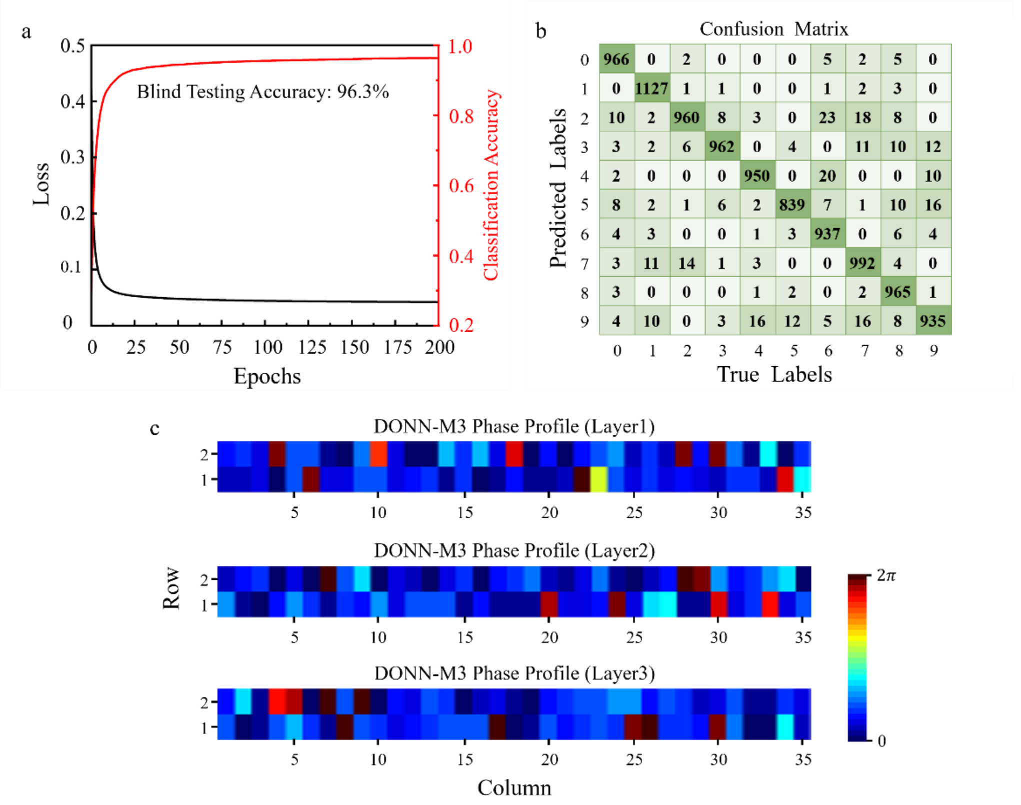

Supplementary Figure 5. Numerical calculation results of on-chip DONN-M3. a Loss curves on the training set (black line) and blind testing accuracy curves on the test set (red line) for the optimized on-chip DONN-M3 during the training procedure. **b** Confusion matrix of the MNIST test set obtained by simulation shows that the accuracy of blinding test set is 96.3%. **c** Phase profile of the on-chip DONN-M3 hidden layer after training, the linear phase profile of each hidden layers is converted to a 2 × 35 matrix image, and each rectangular pixel in the figure represents a pretrained phase value of a neuron.

Supplementary Note 3: Numerical analysis of phase errors

Specific phase values of neurons can be pretrained and obtained in advance, and then the physical connection of these phase values on hardware can be realized through physical structures. However, inevitable errors would be caused in the fabrication process. Eq. (S-6) is the transfer matrix of the on-chip DONNs. Here, we assume that the error factor is $\delta = (\delta_1, \delta_2, \cdots, \delta_m)$, whose values are ranging from 0 to 1. The phase value $Phase^{err}$ including machining errors is the product of the original phase value $Phase^{cal}$ and the error factors, as shown in Eq. (S-8). (S-7) and (S-9) are the diagonal matrixes before and after the fabrication errors are introduced, respectively.

$$Y = W^{L+1}\Phi^{L}W^{L}\cdots W^{2}\Phi^{1}W^{1}X \tag{S-6}$$

$$\Phi^{L} = \begin{bmatrix} e^{j\varphi_{1,1}^{L}} & 0 & \cdots & 0 & 0 \\ 0 & e^{j\varphi_{2,2}^{L}} & \cdots & 0 & 0 \\ \vdots & \vdots & \ddots & \vdots & \vdots \\ 0 & 0 & \cdots & e^{j\varphi_{m-1,m-1}^{L}} & 0 \\ 0 & 0 & \cdots & 0 & e^{j\varphi_{m,m}^{L}} \end{bmatrix} \tag{S-7}$$

$$\begin{cases} Phase^{cal} = \left(\varphi_{1,1}^{L}, \varphi_{2,2}^{L}, \varphi_{3,3}^{L}, \cdots, \varphi_{m,m}^{L}\right) \\ Phase^{err} = \left(\delta_1 \cdot \varphi_{1,1}^{L}, \delta_2 \cdot \varphi_{2,2}^{L}, \delta_3 \cdot \varphi_{3,3}^{L}, \cdots, \delta_m \cdot \varphi_{m,m}^{L}\right) \\ Phase^{err} = \left(\varphi_{1,1}^{L,err}, \varphi_{2,2}^{L,err}, \varphi_{3,3}^{L,err}, \cdots, \varphi_{m,m}^{L,err}\right) \end{cases} \tag{S-8}$$

$$\Phi_{err}^{L} = \begin{bmatrix} e^{j\varphi_{1,1}^{L,err}} & 0 & \cdots & 0 & 0 \\ 0 & e^{j\varphi_{2,2}^{L,err}} & \cdots & 0 & 0 \\ \vdots & \vdots & \ddots & \vdots & \vdots \\ 0 & 0 & \cdots & e^{j\varphi_{m-1,m-1}^{L,err}} & 0 \\ 0 & 0 & \cdots & 0 & e^{j\varphi_{m,m}^{L,err}} \end{bmatrix} \tag{S-9}$$

where X is the input signals, Y is the output results, W^{i} $(i = 1,2,\dots,L+1)$ is the diffractive connection matrix, $Phase^{cal}$ is the pretrained parameters, δ is the error factor generated by the fabrication, $Phase^{err}$ is the product of $Phase^{cal}$ and δ, Φ^{L} and Φ_{err}^{L} are diagonal matrices.

Supplementary Note 4: On-chip DONN-system: fabrication and measurement

Supplementary Note 4.1: Device fabrication of on-chip DONN-I1 and DONN-I3

To experimentally evaluate the performance of the proposed on-chip DONNs, on-chip DONN-I1 and DONN-I3 were fabricated. For example, Fig. S6 shows the micrograph of Chip-3, which contains the on-chip DONN-I3 with three HLs. The input signals are loaded by two cascaded 1 × 2 Multimode interferometers (MMI) and four phase shifters. In this design, the thickness and width of the single-mode waveguide are 220 nm and 450 nm, and the footprint of the on-chip DONN-I1 and DONN-I3 are about 0.3 × 0.5 mm^2 and 0.3 × 1.0 mm^2, respectively. Fig. S7 is a picture of the chip after packaging.

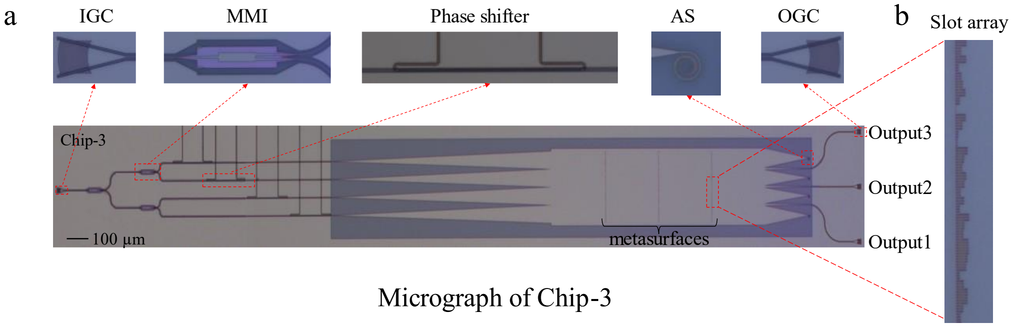

Supplementary Figure 6. Micrograph of Chip-3 and related devices. a Micrograph of Chip-3. The signal is loaded onto the waveguide through the input grating coupler (IGC); then, they are divided into four channels by two cascaded 1 × 2 MMIs. The input phase information is loaded by a thermo-optical phase shifter on each waveguide. After the signal is loaded onto the phase of light, it passes through three HLs (metasurfaces) and enters the output waveguide at the output interface. Finally, it is captured by the optical power meter via the output grating couplers (OGC). AS is the power attenuation structure, which is to prevent the negative impacts of light reflection in the non-detection area of the output interface. **b** Close-up of the HL, in which a silicon slot group consists of three identical slots filled with silicon dioxide to map the phase values onto physical structures.

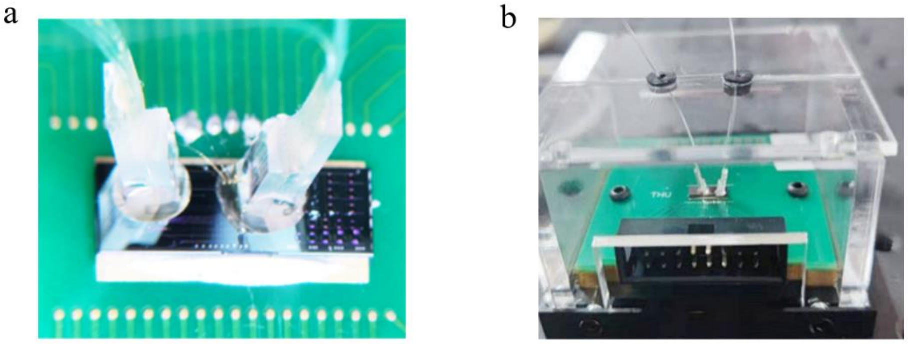

Supplementary Figure 7. Chip package diagram of on-chip DONN. a Close-up of chip wiring and packaging. **b** Diagram after packaging of the on-chip DONN. A protection device is adopted to the encapsulated chip, and the positions of the input and output optical fibers are fixed, which is convenient for the follow-up experimental works.

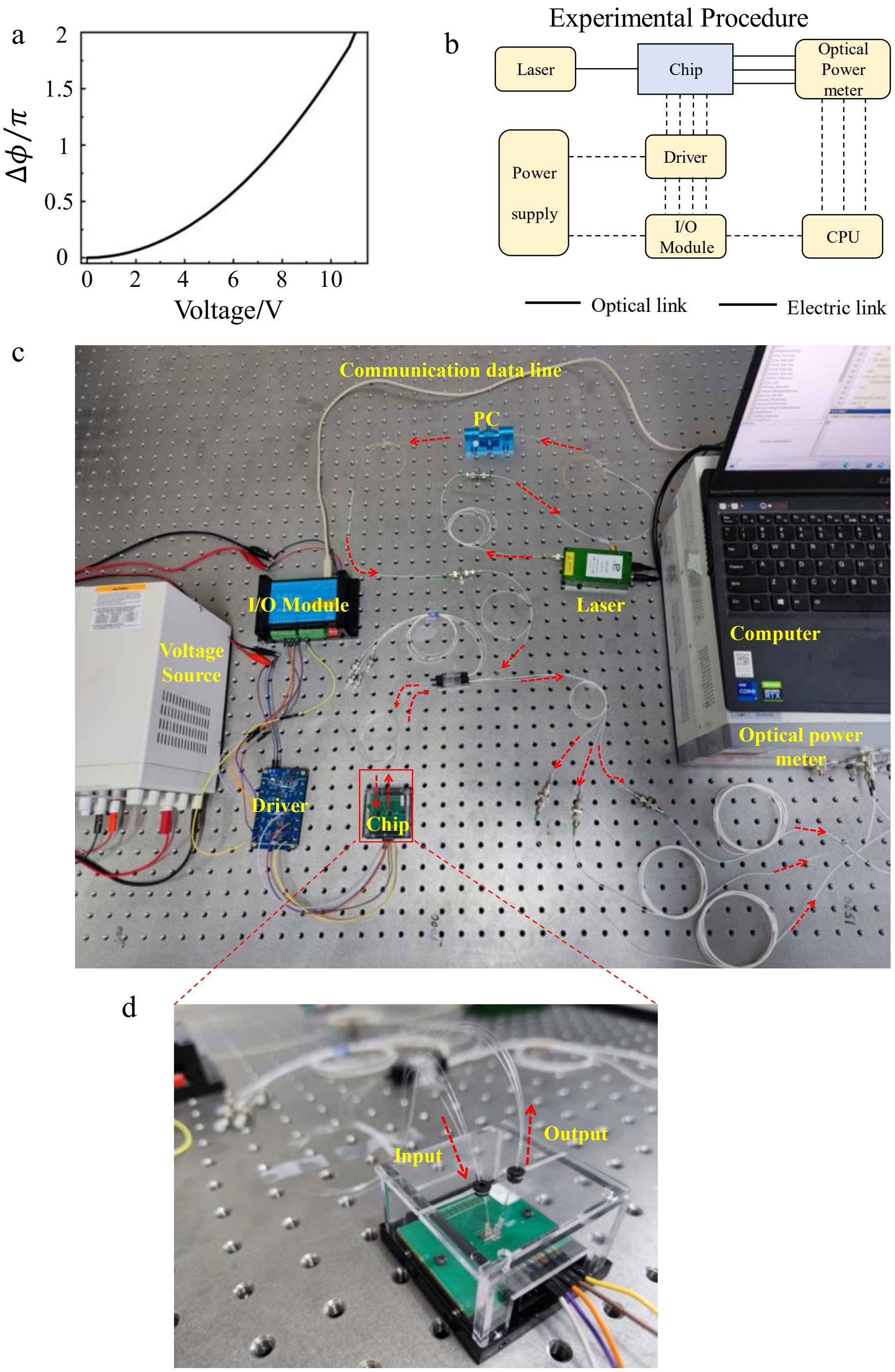

Supplementary Figure 8. Experimental setup and testing process. a Experimentally obtained phase modulation curve as a function of the applied voltage. **b** Experimental testing procedure. **c** Experimental devices. **d** Close-up of the chip after packaging.

Supplementary Note 4.2: Experimental demonstration of on-chip DONN-I1 and DONN-I3

For the Iris plants dataset, each sample has four features, namely calyx length, calyx width, petal length, and petal width. The category of Iris flower species can be identified through these features. In this experiment, the signal is loaded via thermo-optical modulators. The relationship between the voltage and phase curve is shown in Fig. S8a. The experimental testing flow chart for predicting the classification tasks is shown in Fig. S8b. Fig. S8c shows the experimental devices. In addition, due to the fabrication errors, the testing accuracies of the Iris plants dataset of on-chip DONN-I1 and DONN-I3 are 56.7% and 60%, respectively, without introducing the algorithm compensation in experiments; When the algorithm compensation is introduced into the experiments, the testing accuracies of the on-chip DONN-I1 is improved to 86.7%, and DONN-I3 is improved to 90%. Fig. S9 shows the experimental testing results before and after introducing the algorithm compensation.

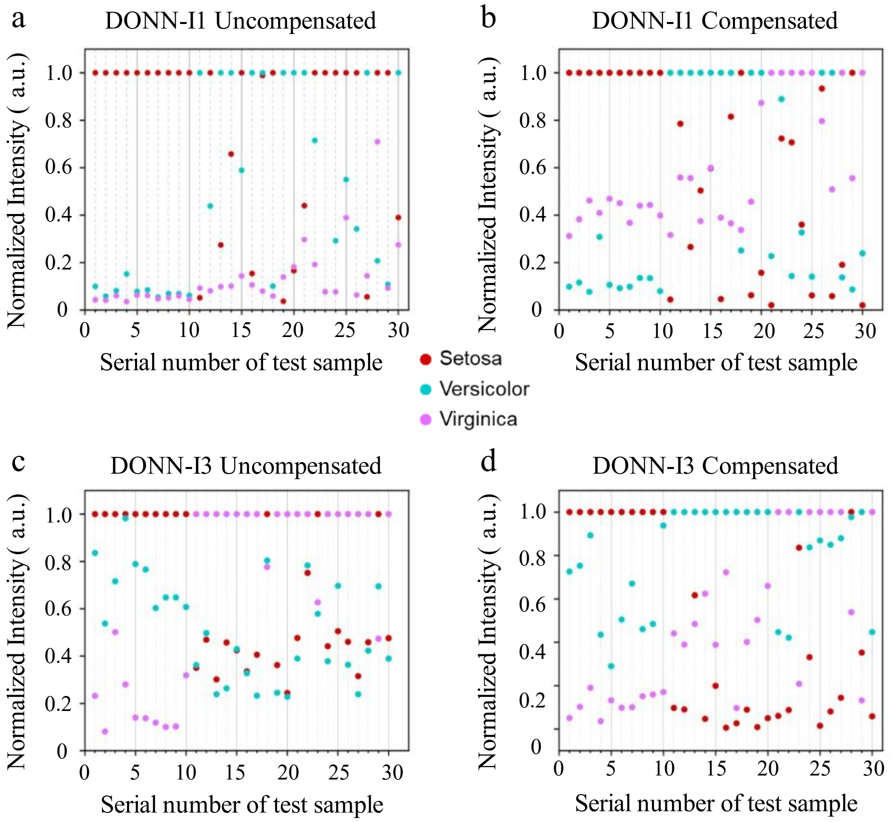

Supplementary Figure 9. Experimental test results of on-chip DONN-I1 and DONN-I3. a, b Experimental testing results of the on-chip DONN-I1, in which the red dot represents “Setosa,” the blue dot represents “Versicolor,” and the purple dot represents “Virginia.” In addition, the correct prediction result of serial numbers 1-10 should be “Setosa,” in other words, the power value corresponding to the red dot should be the maximum. The correct prediction result of serial numbers 11-20 should be “Versicolor,” which means that the power value corresponding to the blue dot should be the largest. The correct prediction result of serial numbers 21-30 should be “Virginia,” at this time, the power value corresponding to the purple dot should be the largest. For example, in Fig. S9a, the power value of the red dot for the No. 5 test sample is the largest, so the prediction is correct; while the power of the blue dot for the No. 15 testing sample should be the largest, but the power of the red dot is the largest at this time, so the prediction is incorrect. **c, d** Testing results of the on-chip DONN-I3, and the data analysis is the same as **a** and **b**. The intensity is normalized according to the specific sample; where, the unit of the normalized power ‘a.u.’ is the abbreviation of ‘arbitrary unit’.

Supplementary Note 4.3: Device fabrication of on-chip DONN-M3

The general fabrication process of the on-chip DONN-M3 is the same as that of on-chip DONN-I1 and DONN-I3. The difference is that the materials of heating electrodes and metal wires are different. The details are introduced in the "Methods" section of the main text. The on-chip DONN-M3 is designed and fabricated for the MNIST classification task. The input 28 × 28 grayscale image is reshaped into a 784 × 1 vector and compressed by the input layer into ten features, thus the fabricated chip has 10 input gratings and 10 output gratings. The input gratings are used for signal loading of 10 features after compression, and the 10 output gratings are used for recognition of handwritten digits (0-9). The microscope and scanning electron microscopy (SEM) images of the on-chip DONN-M3 are shown in Fig. S10a and S10b, respectively. The picture after wiring and packaging of the on-chip DONN-M3 is shown in Fig. S10c, and the experimentally obtained phase modulation curve as a function of the applied voltage is shown in Fig. S10d.

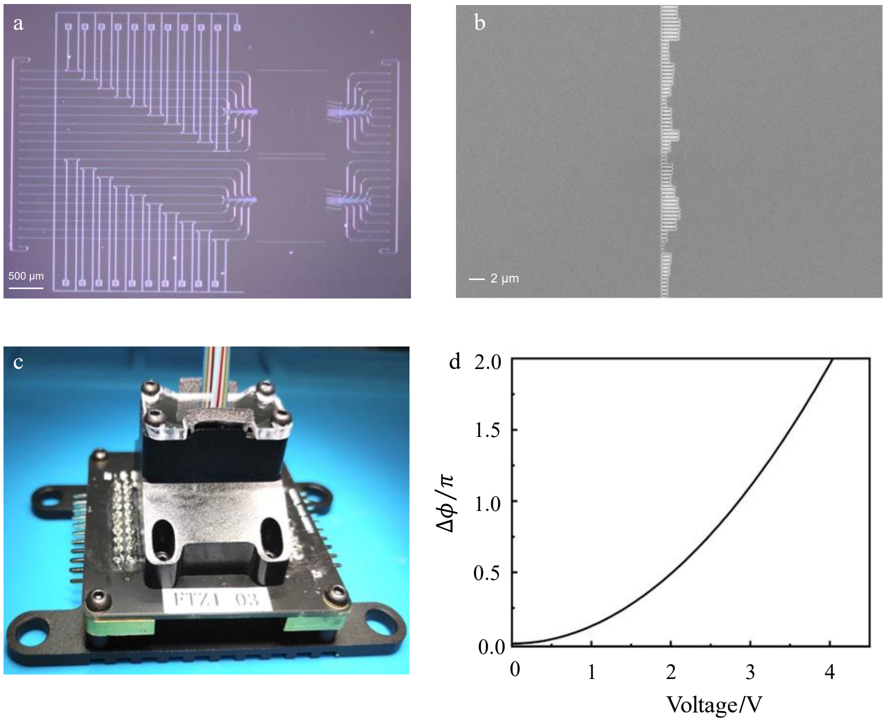

Supplementary Figure 10. Structure diagram and package diagram of the on-chip DONN-M3. a Micrograph of the on-chip DONN-M3, which contains two identical structures. **b** The SEM image of silicon slots structure in the on-chip DONN-M3, the structure before the deposition of silicon dioxide protective layer. **c** Diagram of the DONN chip after packaging. **d** Experimentally obtained phase modulation curve as a function of the applied voltage.

Supplementary Note 5: Compensation analysis of on-chip DONN errors

Supplementary Note 5.1: System error compensation for the Iris flower classifier

Here is a specific example for the compensation analysis of on-chip DONN errors, Fig. S11a is a 1-hidden-layer DONN. Fig. S11b is the logic diagram of Fig. S11a, and Fig. S11c is the equivalent logic diagram after introducing the experimental compensation algorithm. The mathematical calculation process of Fig. S11a is expressed by Eq. (S-10). When the inevitable errors are brought by the signal loading and fabrication process, the mathematical calculation process is expressed by Eq. (S-11). To reduce the impacts of signal loading and machining errors on the performance of on-chip DONNs, two steps are adopted to reduce the negative influences caused by the signal loading and machining errors. First, the phase compensation is introduced in the signal loading stage, meanwhile, the compensated phases of $(\varphi_{11}, \varphi_{22}, \varphi_{33}, \varphi_{44})$ are generated by the voltages of $(\Delta V_1, \Delta V_2, \Delta V_3, \Delta V_4)$ according to Fig. S8a. Then, in the signal detection stage, the output power of different ports is multiplied by a power compensation factor, which refers to Fig. S11c for the specific process. Among them, the phase compensation process in the first stage is depicted by Eq. (S-12). The power detection process is depicted by Eq. (S-13), and the power compensation stage is expressed by Eq. (S-14).

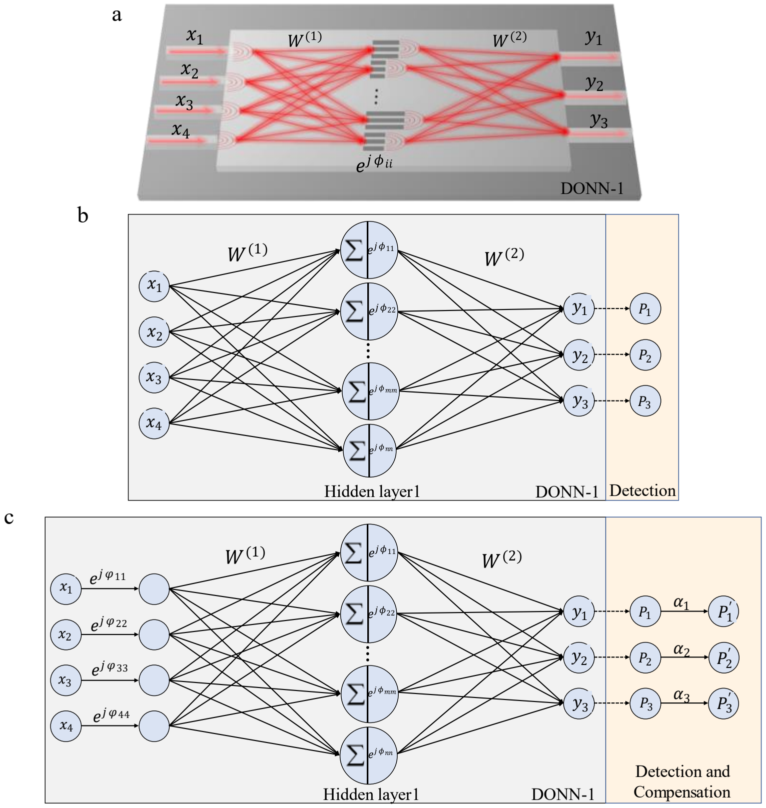

Supplementary Figure 11. On-chip DONN calculation process and its error compensation process. a On-chip DONN with one hidden layer. **b** Logic diagram of Fig. S11a. **c** Equivalent logic diagram of Fig. S11b after introducing the experimental compensation algorithm.

$$\begin{bmatrix} y_1 \\ y_2 \\ y_3 \end{bmatrix} = \begin{bmatrix} W^{(2)} \end{bmatrix}_{3\times m}^{T} \begin{bmatrix} e^{j\phi_{11}} & 0 & 0 & \cdots & 0 \\ 0 & e^{j\phi_{22}} & 0 & \cdots & 0 \\ \vdots & \ddots & \ddots & \ddots & \vdots \\ 0 & \cdots & 0 & \ddots & 0 \\ 0 & \cdots & 0 & 0 & e^{j\phi_{mm}} \end{bmatrix} \begin{bmatrix} W^{(1)} \end{bmatrix}_{m\times 4}^{T} \begin{bmatrix} x_1 \\ x_2 \\ x_3 \\ x_4 \end{bmatrix} \quad \text{(S-10)}$$

$$\begin{bmatrix} y_1 \\ y_2 \\ y_3 \end{bmatrix} = \begin{bmatrix} W^{(2)} \end{bmatrix}_{3\times m}^{T} \begin{bmatrix} e^{j\left(\phi_{11}+\phi_{11}^{err}\right)} & 0 & 0 & \cdots & 0 \\ 0 & e^{j\left(\phi_{22}+\phi_{22}^{err}\right)} & 0 & \cdots & 0 \\ \vdots & \ddots & \ddots & \ddots & \vdots \\ 0 & \cdots & 0 & \ddots & 0 \\ 0 & \cdots & 0 & 0 & e^{j\left(\phi_{mm}+\phi_{mm}^{err}\right)} \end{bmatrix} \begin{bmatrix} W^{(1)} \end{bmatrix}_{m\times 4}^{T} \begin{bmatrix} x_1 \\ x_2 \\ x_3 \\ x_4 \end{bmatrix} \quad \text{(S-11)}$$

$$\begin{bmatrix} y_1 \\ y_2 \\ y_3 \end{bmatrix} = \begin{bmatrix} W^{(2)} \end{bmatrix}_{3\times m}^{T} \begin{bmatrix} e^{j\phi_{11}^{err}} & 0 & 0 & \cdots & 0 \\ 0 & e^{j\phi_{22}^{err}} & 0 & \cdots & 0 \\ \vdots & \ddots & \ddots & \ddots & \vdots \\ 0 & \cdots & 0 & \ddots & 0 \\ 0 & \cdots & 0 & 0 & e^{j\phi_{mm}^{err}} \end{bmatrix} \begin{bmatrix} W^{(1)} \end{bmatrix}_{m\times 4}^{T} \begin{bmatrix} e^{j\varphi_{11}} & 0 & 0 & 0 \\ 0 & e^{j\varphi_{22}} & 0 & 0 \\ 0 & 0 & e^{j\varphi_{33}} & 0 \\ 0 & 0 & 0 & e^{j\varphi_{44}} \end{bmatrix} \begin{bmatrix} x_1 \\ x_2 \\ x_3 \\ x_4 \end{bmatrix} \quad \text{(S-12)}$$

$$\begin{bmatrix} P_1 \\ P_2 \\ P_3 \end{bmatrix} = \begin{bmatrix} |y_1|^2 \\ |y_2|^2 \\ |y_3|^2 \end{bmatrix} \quad \text{(S-13)}$$

$$\begin{bmatrix} P_1' \\ P_2' \\ P_3' \end{bmatrix} = \begin{bmatrix} \alpha_1 P_1 \\ \alpha_2 P_2 \\ \alpha_3 P_3 \end{bmatrix} = \begin{bmatrix} \alpha_1 & 0 & 0 \\ 0 & \alpha_2 & 0 \\ 0 & 0 & \alpha_3 \end{bmatrix} \begin{bmatrix} P_1 \\ P_2 \\ P_3 \end{bmatrix} \quad \text{(S-14)}$$

For the phase compensation stage, a set of optimized fixed voltage values$(\Delta V_1, \Delta V_2, \Delta V_3, \Delta V_4)$ can be found through the corresponding algorithm. When this set of fixed voltage values is found, phase compensation can be realized by adding the original signal voltage value to this set of voltage values; For the power compensation stage, based on the phase compensation result, a set of optimized fixed coefficients $(\alpha_1, \alpha_2, \alpha_3)$ can be found through the optimization search algorithm. Finally, by multiplying the output power of all samples by the set of fixed power coefficients, the whole error compensation stage can be completed. P_1, P_2 and P_3 represent the optical power detected by different output ports respectively.

Supplementary Note 5.2: System error compensation for the Handwritten digit classifier

The dimension of the MNIST dataset is relatively high, thus the compensation method for online in-situ training would take a long time even if 100 test sets are selected. However, based on the practical existing experimental conditions, the stability of the experimental system cannot be guaranteed for a long time, due to the optical path would be affected by polarization, ambient temperature, and vibration during the signal loading stage. Thus, the online in-situ training compensation would be difficult. Therefore, we chose the second part of the compensation method introduced in Supplementary Note 5.1 as shown in Eq. (S-14)

for getting the error compensation factors offline. The MNIST classification task is more complex than the iris classification task, the system errors are more difficult to be corrected, thus a 10×10 full connection layer (W^{full}) after the DONN-M3 chip is adopted to compensate the system errors, and the mathematical expression of the compensation process is shown in Eq. (S-15). The full connection layer can be obtained through machine learning. When the training is completed, the full connection layer is fixed in the process of the blind test sets classification.

$$\begin{bmatrix} P_1' \\ P_2' \\ \vdots \\ P_{10}' \end{bmatrix} = \left[W^{full}\right]^T_{10\times10} \begin{bmatrix} P_1 \\ P_2 \\ \vdots \\ P_{10} \end{bmatrix} = \begin{bmatrix} w_{11} & \cdots & w_{1,10} \\ \vdots & \ddots & \vdots \\ w_{10,1} & \cdots & w_{10,10} \end{bmatrix}^T_{10\times10} \begin{bmatrix} P_1 \\ P_2 \\ \vdots \\ P_{10} \end{bmatrix} \quad \text{(S-15)}$$

Supplementary Note 6: Algorithm compensation

To compensate for the fabrication errors, an algorithm compensation method, including phase compensation and power compensation, is used to reduce the negative impacts of the introduced errors. Here, the input signals were applied to the phases of light through the input voltages, and the phase modulation curve is shown in Fig. S8a.

Supplementary Note 6.1: Parameters of the algorithm compensation process

X_0: Original voltage configurations of the input signals (without compensation);
X: Compensation voltage for optimization (Particles' positions);
X_{min}: Minimum compensation voltage;
X_{max}: Maximum compensation voltage;
V: Updating speed of X (Particles' velocities);
V_{min}: Minimum updating speed of X for optimization;
V_{max}: Maximum updating speed of X for optimization;
T: True labels;
Y: Chip's outputs;
$Chip(\cdot)$: Chip's forward calculation;
$Max\_iteration$: Maximum number of iterations;
E: Targeted average loss;
N_p: Number of particles;
N_s: Number of samples in training process;
i: Iteration index;
j: Particle index;
k: Sample index;
α: Power compensation factor for optimization;
p: Searching index of power compensation factor;
$\Delta\alpha$: Searching step of power compensation factor;
α_{num}: The total number of the searching index;
$Loss(\cdot)$: Loss function of the calculation process between Y and T;
$Ncc(\cdot)$: Counting when the outputs Y classify correctly with respect to T;
L_s: Single loss for each sample;
L_{pavg}: Average loss for each particle;
N_c: The total number of samples which were classified correctly;
A_{cavg}: Accuracy after power compensation;
P_{best}: The best performance for the particles through training;

G_{best}: The best performance for the whole particle group through training;
L_{Gbest}: The average loss of G_{best} configurations;
$Sort\ (\cdot)$: Picking the best configurations from the samples;
$Find\ (\cdot)$: Finding the corresponding voltage compensation configurations with respect to the power compensations.
$Generate\ (\cdot)$: Generating the V in the next iteration for particles.

Supplementary Note 6.2: Pseudo code of the optimization process

Error compensation is an optimization problem, which aims to minimize the impact of errors on the performance of the system. The optimization problem is described mathematically as Eq. (S-16).

$$\underset{X,a}{argmin} \sum_{dim(T)} \left\{ \left[Chip(X_0 + X) \odot a \right] - T \right\}^2 \tag{S-16}$$

To find the minimum value of the optimization object function above, two steps are required:
Step 1. Compensations on voltages by fixing α=1 to make the loss function which is described as Eq. (S-17) to be converged, the compensation in this stage is called phase compensation, where $\odot$ indicates multiplication with the corresponding element.

$$\sum_{dim(T)} \left\{ Chip(X_0 + X) - T \right\}^2 \tag{S-17}$$

In this compensation, the optimized best voltage compensation configurations in each iteration will become the candidates for the power compensation in the next step.
Step 2. Compensation on power by fixing the best voltage compensation configurations in each iteration of the previous, $X = (\Delta V_1, \Delta V_2, \Delta V_3, \Delta V_4)$, and then finding a set of optimal power compensation factors $\alpha = (\alpha_1, \alpha_2, \alpha_3)$ by the traversal search method to further increase the accuracy of classification.

Before the algorithm starts, several determinations are required as:
① Determine X_{max}, X_{min}, V_{max}, V_{min}, $Max\_iteration$, N_p, etc.;
② Randomly generate X for each particle;
③ Set P_{best}, G_{best}, L_{Gbest} and α_{best} to be the positive infinity.

Then, start the training process: [X_{best}, α_{best}] = **Function** training (X_0, T).

while ($i < Max\_iteration$ **and** $L_{Gbest} <= E$):

for ($j = 1; j <= N_p; j{+}{+}$):

$L = 0;$

for ($k = 1; k <= N_s; k{+}{+}$):

$Y = Chip(X_0(k) + X(i, j))$;

$L = L + Loss(Y, T(k));$

end

$L_{pavg}(i,j) = L/N_s;$

$\boldsymbol{if}\ L_{pavg}(i,j) < P_{best}(j)$:

$P_{best}(j) = L_{pavg}(i,j)$;

end

if $Sort(P_{best}) < G_{best}(i)$:

$G_{best}(i) = Sort\ (P_{best})$;

end

$V = Generate\ (V, X, P_{best}, G_{best})$;

$X = X + V$;

$i = i + 1$;

end

for $(i = 1; i < length\ (G_{best}); i++)$

for $(p = 1; p < \alpha_{num}; p++)$

$N_c = 0$;

for $(k = 1; k < N_s; k++)$

$Y = Chip\ (X_0(k) + X_{Gbest}(i)) \cdot \alpha(p)$;

$N_c = N_c + N_{cc}(Y, T(k))$;

end

$A_{cavg}(i,p) = A_c/N_s$;

end

end

$\alpha_{best} = Sort\ (A_{cavg})$;

$X_{best} = Find\ (\alpha_{best})$;

Validating performances using X_{best} and α_{best} on testing set.

return X_{best} and α_{best}

Supplementary Note 6.3: Optimization flow chart

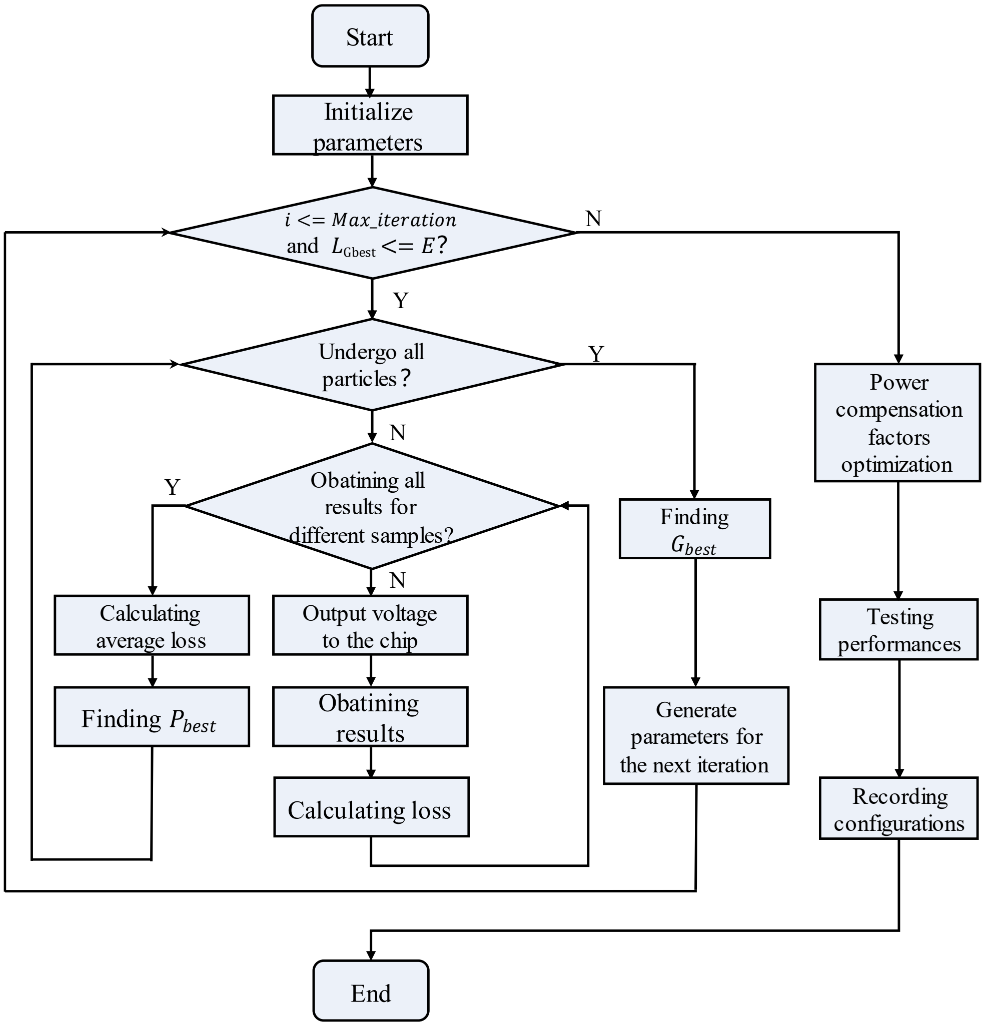

Supplementary Figure 12. The flow chart of algorithm compensation process.

Supplementary Note 6.4: Brief introduction of the compensation algorithm

We propose an algorithm compensation method including two-stage of phase compensation and power compensation. Through training, the optimized candidate voltage values in the phase compensation stage are notated as X, and power compensation factors in the power compensation stage are noted as α. Therefore, the optimization objective function of this optimization problem can be expressed as Eq. (S-16).

In the phase compensation stage, the aim is to find the minimum value of loss function in Eq. (S-17). Meanwhile, the X_0 represents the original voltage configurations, the operator dim () means taking the number of dimensions of the vector, and T should be the one-hot vector of the original labels from the dataset. This training stage is based on particle swarm optimization (PSO). From the very beginning of the algorithm, parameters regarding iterations such as the maximum iteration numbers and the minimum values of the loss function are needed to be set. Then, configurations related to particles such as the ranging boundary of voltages (particles' positions), and the boundary of the updating speed (particles'

velocities), should be determined. Other parameters related to the compensation algorithm should also be set. Then, this algorithm will randomly generate the group of compensation voltage configurations particle by particle. Afterwards, the algorithm will run into iterations to find the candidate X which can obtain the lowest value of the loss function.

As can be vividly seen from Fig. S12, before the beginning of the i-th iteration, the compensation voltage configurations for each particle are generated based on the best particle performance and other statistical features extracted from all particles in the (i-1)-th iteration. The footnote j represents the particle's index. If i is lower than the maximum iteration number or the loss value of (i-1)-th iteration is larger than the minimum loss value, the algorithm will operate into the i-th iteration. Otherwise, the whole iteration will expire.

In the i-th iteration, performances of different particles' compensation voltage configurations on different samples from the training dataset are needed to be recorded. For each compensation voltage configuration of one particle, PC will add the X into the original voltage of each sample from the training dataset. Then, these modified voltages will be sent onto the phase-shifter via the I/O module and the driver (Fig. S8). The intensities of the output channels Y without power compensation are transmitted back to the central processing unit (CPU) to calculate the loss values for each sample.

If voltages of all samples are sent, then the algorithm runs into the evaluation of particles' performances. For each particle, the average loss can be computed by averaging the single loss value for each sample. After all particles' performances are evaluated, the best performance can be obtained from the best performance of each particle for the lowest loss. The corresponding value is called the group best. The iteration will be executed and all results and voltage configurations will be recorded in each iteration until either iteration's index exceeds the maximum value or the loss value is lower than the targeted value. The recorded group best configurations in each iteration will participate in the power compensation stage.

In the power compensation stage, power compensation factors are adopted via the traversal method to further increase the classification accuracy. By pre-determining both maximum and minimum power compensation factor's boundary with searching steps, one can record the accuracies with respect to different power compensation factors and voltage configurations from each iteration's G_{best} in the phase compensation stage, and the best power compensation factor can be found according to the maximum accuracy.

In the end, this algorithm compensation method can find the best X and α for the minimum value of the target function and achieve the best prediction accuracy. Additionally, other algorithms can also be considered for experimental error compensation, such as the compensation algorithms used in the previous works [4,5].

Supplementary Note 7: On-chip DONN computational speed and power consumption

Supplementary Note 7.1: Computation speed

By assuming that an on-chip DONN system has N nodes and a 100 GHz data detection rate, the system includes m layers, while each layer contains an $N \times N$ weighting matrix, the number of floating-point operations per second (FLOPS) can be evaluated by the Eq. (S-18) [6]. Taking the on-chip DONN-I3 we have already fabricated as an example, here $m = 2$, $N = 186$, therefore, the computing speed of the DONN-I3 can reach 1.38×10^{16} FLOPS.

$$R = 2m \times N^2 \times 10^{11} \text{ FLOPS} \tag{S-18}$$

For computational speed, in addition to numbers of operations per second, the latency required to complete a single task is also critical. For the latency of on-chip DONNs, it is defined as the overall time between the start of the signal loading and the detection of the output, in other words, the process does not include input signal loading time and output detection time [1,7]. In our on-chip DONN, the latency can be calculated by Eq. (S-19):

$$T_{delay} = D_1 \cdot \left(\frac{c_0}{n_{eff1}} \right)^{-1} + M \cdot D_m \cdot \left(\frac{c_0}{n_{eff2}} \right)^{-1} + \left[D_2 + (M-1) \cdot D_p + D_3 \right] \cdot \left(\frac{c_0}{n_{eff3}} \right)^{-1} \quad \text{(S-19)}$$

where D_1 is the distance from the narrow waveguide to the slab waveguide; c_0 is the vacuum light speed, n_{eff1} is the ERI of the narrow waveguide; M is the total number of the hidden layers; D_m is the length of the longest slot in the hidden layers; n_{eff2} is the ERI of the silicon slots filled with silicon dioxide; D_2 is the distance from the start of the slab waveguide to the first hidden layer; D_p is the propagation distance between the hidden layers; D_3 is the propagation distance between the last hidden layer and the output layer; n_{eff3} is the ERI of the slab waveguide. As an example, for our designed Chip-3, $n_{eff1} = 2.33$, $n_{eff2} = 2.166$, $n_{eff3} = 2.84$, the latency is approximately 27.56 ps. The relevant parameters in Eq. (S-19) have been annotated in the chip micrograph as shown in Fig. S13.

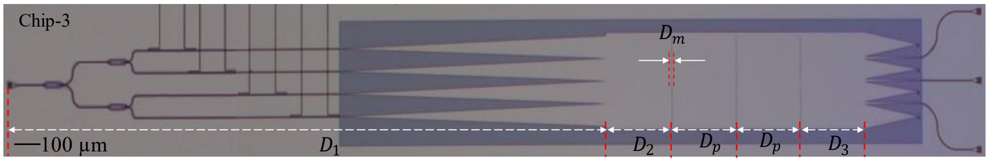

Supplementary Figure 13. The micrograph of Chip-3. The relevant parameters in Eq. (S-19) are marked in the figure.

Supplementary Note 7.2: Power consumption

First, the input power of the laser under 1.55 μm is 32 mW. The input signal is loaded by the thermo-optic phase shifters, and the average energy required to set each phase shifter a 2π rad is approximately 30 mW. In addition to the light source and the signal loading stage, the calculation process of the computing part in our proposed on-chip DONN is fully passive, so the system does not need to consume extra energy in processing the inference tasks. Therefore, in terms of theoretical calculation, the energy consumed to complete one calculation for the proposed on-chip DONN-I3 system is about 1.1×10^{-17} J/FLOP. Moreover, to reduce the power consumption of the phase shifters, one could use doped side heaters [8,9] or liquid-crystal-based phase shifters [10].

In addition, for the on-chip DONN system, the power consumption of the metasystem is the summation of the power required for propagation and the optical power required to support an optical nonlinearity that could be potential implementations of future devices. Here, the transmission loss of light in the working process of on-chip DONN system is ignored, and power consumption is mainly required to support an optical nonlinearity. Therefore, assume the saturation power for our saturable absorber is p MW/cm^2 ($p \approx 1, \mathrm{e.g.\,graphene}$), and an area of a neuron $A = 1.5\ \mu m \times 2.0\ \mu m$, then the total power needed to run the system is estimated to be $P = p \times A \times N = 3N(mW)$, meanwhile, N is the neuron number per layer. For the proposed on-chip DONN-I3 system ($N = 186$), the input power of the laser is approximately 32 mW and the power required to maintain the operation of the phase shifter is 4×30 mW, the operations per second is $R = 1.38 \times 10^{16}$ FLOPS, thus the power consumption is approximately 5.15×10^{-17} J/FLOP.

Supplementary Note 8: Comparison of partial performances of different optical neural networks (ONNs)

Theoretically, we compare the number of neurons integrated per square millimeter, the total number of operands processed per square millimeter per second, and the energy consumed by each operand after the introduction of nonlinearity. The specific calculation results are shown in Supplementary Table 3.

Supplementary Table 3 | Comparison of partial performances of different ONNs

Index / Works	Footprint (mm^2)	Throughput (TOPS)	Integration in theory (NBUs/ mm^2)	Computing capacity in theory (FLOPS/mm2)
Ref. [6]	0.68	6.4	< 10	9.41×10^{12}
Ref. [11]	0.36	21.6	< 10	6.0×10^{13}
Ref. [12]	1.2	32.0	< 20	2.67×10^{13}
Ref. [13]	2.12	30.0	< 25	1.42×10^{13}
Ref. [14]	6.07	28.8	< 5	1.8×10^{12}
Our work DONN-I3	0.3	1.38×10^4	$\sim 2 \times 10^3$	4.6×10^{16}

In Supplementary Table 3, TOPS: Trillions (10^{12}) of operations per second, NBUs: Number of the basic units, FLOPS: the number of floating-point operations per second. For the throughput, which are uniformly calculated according to Eq. (5).

Supplementary Note 9: Comparison of on-chip DONN-I3 with other research works and commercial products in terms of Throughput and Operational power consumption

Supplementary Table 4 | Comparison of on-chip DONN-I3 with other research works and commercial products

Index / Works	Throughput (TOPS)	Operational power (J/FLOP)
Ref. [6]	6.4	7.66×10^{-14}
Ref. [11]	21.6	2.14×10^{-13}
Ref. [12]	32	1.41×10^{-15}
Ref. [13]	30	3.07×10^{-14}
Ref. [14]	28.8	5.9×10^{-15}
Google TPU [15]	23	2.15×10^{-13}
NVIDIA Tesla T4 [16]	130	5.4×10^{-13}
HUAWEI Ascend 910 [17]	640	5.45×10^{-13}
Our work (DONN-I3)	1.38×10^4	1.1×10^{-17}

In Supplementary Table 4, TOPS: Trillions (10^{12}) of operations per second, FLOP: floating-point operations.

Supplementary References

1. Fu, T.Z. *et al.* On-chip photonic diffractive optical neural network based on a spatial domain electromagnetic propagation model. *Optics Express* **29**, 31924-31940 (2021).
2. Lin, X. *et al.* All-optical machine learning using diffractive deep neural networks. *Science* **361**, 1004-1008 (2018).
3. Wang, Z. *et al.* On-chip wavefront shaping with dielectric metasurface. *Nature Communications* **10**(2019).
4. Zhou, T.K. *et al.* Large-scale neuromorphic optoelectronic computing with a reconfigurable diffractive processing unit. *Nature Photonics* **15**, 367-373 (2021).
5. Bandyopadhyay, S., Hamerly, R. & Englund, D. Hardware error correction for programmable photonics. *Optica* **8**, 1247-1255 (2021).
6. Shen, Y.C. *et al.* Deep learning with coherent nanophotonic circuits. *Nature Photonics* **11**, 441-446 (2017).

7. Zarei, S., Marzban, M.R. & Khavasi, A. Integrated photonic neural network based on silicon metalines. *Optics Express* **28**, 36668-36684 (2020).
8. Absil, P.P. *et al.* Silicon photonics integrated circuits: a manufacturing platform for high density, low power optical I/O's. *Optics express* **23**, 9369-9378 (2015).
9. Masood, A. *et al.* Comparison of heater architectures for thermal control of silicon photonic circuits. in *10th International Conference on Group IV Photonics* 83-84 (IEEE, 2013).
10. Xing, Y. *et al.* Digitally controlled phase shifter using an SOI slot waveguide with liquid crystal infiltration. *IEEE Photonics Technology Letters* **27**, 1269-1272 (2015).
11. Zhang, H. *et al.* An optical neural chip for implementing complex-valued neural network. *Nature Communications* **12**, 1-11 (2021).
12. Zhu, H.H. *et al.* Space-efficient optical computing with an integrated chip diffractive neural network. *Nature Communications* **13**, 1044 (2022).
13. Zhao, X. *et al.* On-chip Reconfigurable Optical Neural Networks. (2021).
14. Feldmann, J. *et al.* Parallel convolutional processing using an integrated photonic tensor core (vol 589, pg 52, 2021). *Nature* **591**, E13-E13 (2021).
15. Jouppi, N.P. *et al.* In-Datacenter Performance Analysis of a Tensor Processing Unit. *44th Annual International Symposium on Computer Architecture (Isca 2017)*, 1-12 (2017).
16. NVIDIA. T4 tensor core datasheet. https://www.nvidia.com/en-us/data-center/tesla-t4/ (accessed November 30, 2021).
17. HUAWEI. A910 AI processor datasheet. https://e.huawei.com/cn/products/cloud-computing-dc/atlas/ascend-910 (accessed November 30, 2021).
